# Histone posttranslational modifications predict specific alternative exon subtypes in mammalian brain

**DOI:** 10.1371/journal.pcbi.1005602

**Published:** 2017-06-13

**Authors:** Qiwen Hu, Eun Ji Kim, Jian Feng, Gregory R. Grant, Elizabeth A. Heller

**Affiliations:** 1 Department of Systems Pharmacology and Translational Therapeutics, University of Pennsylvania, Philadelphia, PA, United States of America; 2 Institute for Translational Medicine and Therapeutics, University of Pennsylvania, Philadelphia, PA, United States of America; 3 Department of Biological Science, Florida State University, Tallahassee, FL, United States of America; 4 Department of Genetics, University of Pennsylvania, Philadelphia, PA, United States of America; 5 Penn Epigenetics Institute, Perelman School of Medicine, University of Pennsylvania, Philadelphia, PA, United States of America; Ottawa University, CANADA

## Abstract

A compelling body of literature, based on next generation chromatin immunoprecipitation and RNA sequencing of reward brain regions indicates that the regulation of the epigenetic landscape likely underlies chronic drug abuse and addiction. It is now critical to develop highly innovative computational strategies to reveal the relevant regulatory transcriptional mechanisms that may underlie neuropsychiatric disease. We have analyzed chromatin regulation of alternative splicing, which is implicated in cocaine exposure in mice. Recent literature has described chromatin-regulated alternative splicing, suggesting a novel function for drug-induced neuroepigenetic remodeling. However, the extent of the genome-wide association between particular histone modifications and alternative splicing remains unexplored. To address this, we have developed novel computational approaches to model the association between alternative splicing and histone posttranslational modifications in the nucleus accumbens (NAc), a brain reward region. Using classical statistical methods and machine learning to combine ChIP-Seq and RNA-Seq data, we found that specific histone modifications are strongly associated with various aspects of differential splicing. H3K36me3 and H3K4me1 have the strongest association with splicing indicating they play a significant role in alternative splicing in brain reward tissue.

## Author summary

The mammalian brain responds to environmental stimuli through changes in gene expression. Over the past decade a robust body of bioinformatics data has shown that in neuronal tissue such gene expression is regulated by changes in the epigenetic landscape, including modifications to chromatin. Further, a small but compelling body of literature has recently described chromatin-regulated alternative splicing, suggesting a novel function for neuroepigenetic remodeling in alternative isoform expression. However, the extent of the genome-wide association between particular histone modifications and alternative splicing remains unclear, in part due to limitation in methods to model the convergence of chromatin modifications and gene expression changes. We report here our innovative computational approach to model the association between alternative splicing and histone marks in the nucleus accumbens, a brain reward region. We found, remarkably, that specific histone marks are associated with and predict both alternative splicing exon type and alternative splicing exon complexity in the brain, with particular histone marks showing the significant enrichment at alternative exons. This approach is the first to model chromatin-mediated alternative splicing globally, and our findings are consistent with recent data on the molecular biology of this mechanism, homing in on a subset of chromatin modifications that are functionally relevant to alternative splicing *in vivo*.

## Introduction

Classic experiments in cell culture systems have defined a ‘histone code’, whereby distinct combinations of histone posttranslational modifications (histone marks) are associated with transcriptional repression or activation and play important roles in many other biological processes [1,2]. Recent evidence indicates that some histone marks, such as H3K36me3 and H3K4me3 are functionally coupled to alternative splicing [3–5], however, a globally systematic analysis to investigate this relationship is lacking. Genome-wide mapping of histone modifications in many organisms has revealed their non-random distribution around exons, with some types of histone marks enriched in exonic regions compared to intronic regions [6,7].

Specifically, several studies on human-derived datasets find that histone modifications are associated with exon inclusion or exclusion, indicating the role of histone modifications in the regulation of alternative splicing. For example, a global analysis of the relationship between alternative splicing and histone modifications in two human cell lines found that histone modifications are globally associated with exon inclusion/exclusion patterns and that the change of histone modification patterns corresponds to cell-type specific exon usage [8]. Additional studies have demonstrated a functional correlation between specific histone marks and splicing outcomes at the human *FgfII* locus [4] and a predictive function for specific histone marks in alternative exon expression in human somatic cells [9]. Table 1 contains a detailed summary of published data on the link between histone modifications and alternative splicing.

**Table 1:** Summary of the link between histone modifications and alternative splicing from published studies.

Model	Cell line/tissue	Main finding	Ref.
Human	H1, IMR90	H3K36me3 significantly enriched in included exons. H3K4me3, H2BK12ac, H4K5ac significantly enriched in excluded exons	[8]
Human	PNT2s, hMSCs	H3K36me3 enrichment leads to exon exclusion at *FGFR2* and this hPTM interacts with spliceosome machinery; H3K4me3 enrichment leads to exon inclusion at *FGFR2*	[4]
Human	CD4+ cell, IMR90 cell, GM12878, K562, H1 hESC and Hep G2	H3K36me3, H3K4me1, H3K4me2, H3K4me3, H4K20me1, H3K27me3, H3K79me1, H3K79me2 enriched in different regions of alternative exons. H3K9me3 is not associated with alternative splicing	[10]
Human	Gm12878, K562 and H1-hESC	H3K36me3, H3K9me3, H4K20me1 and H3K27me3 significantly associated with cassette exon inclusion	[11]
Human	CD4+ T cells	H3K36me3 and H4K20me1 and gene expression directly interact with cassette exon expression	[9]
Human	CD4+ T cell	H3K36me3, H2BK5me1 and H4K20me1 were associated with exon inclusion level	[12]
Human	Gm12878, Hsmm, Huvec, Hepg2, Helas3, K562, H1hesc, Nhek, Nhlf	H3K36me3 was enriched associated with exon inclusion rate. H3K4me1, H3K4me2, H3K4me3, H3K9ac, H3K27ac, H3K79me2, and H2az were positive associated with transcription start-site switching.	[13]
Human	CD4+ T cells	H3K36me3 enrichment correlates with alternative splicing	[14]
Mouse	nucleus accumbens	H3K4me3, H3K36me3, H3K9me3 and H3K27me3 were differentially enriched by cocaine treatment; cocaine treatment correlated alternative isoform expression	[15]

Despite these recent advances, the genome-wide association between histone modifications and alternative splicing, as elucidated by the analysis of global exon inclusion/exclusion level splicing patterns remains unclear. Furthermore, published approaches are not directly applicable to unannotated alternatively spliced exons because the models rely on defined exon and splice isoform annotation. Thus, the goals of our analysis are to define the association of specific alternative splicing patterns and histone modifications and to determine which marks are likely to play a dominant role in the regulation of alternative pre-mRNA splicing, as assessed by the statistically significant associations of these phenomena. In particular, we focused on sequencing from the nucleus accumbens (NAc), a brain-reward region, given evidence that drugs of abuse as well as natural rewards regulate expression of the myriad of enzymes that catalyze and metabolize histone modifications as well as their genome-wide deposition. Indeed, major efforts in next generation chromatin immunoprecipitation sequencing (ChIP-Seq) and RNA-Sequencing (RNA-Seq) of reward brain regions have demonstrated that regulation of the epigenetic landscape likely underlies chronic drug abuse and addiction [16,17]. Furthermore, studies have implicated dysregulation of alternative splicing in human neurological disease [18] and cocaine exposure in mice. Prior studies in mice, including our own, intriguingly revealed that in addition to global regulation of histone modifications [15,16,19], cocaine drives differential alternative splicing to a far greater extent than had been previously appreciated [15]. In addition, our previous study found that cocaine drives enrichment of several histone modifications, including H3K4me3, H3K36me3, H3K9me3 and H3K27me3, on different types of alternatively spliced exons. To expand upon this previous finding, we developed a systematic approach to test for the global association between histone modifications and specific types of alternative splicing.

Given mounting evidence for a role of histone modifications in alternative isoform expression, we developed two independent analyses, which we termed, “Exon Alternative Splicing Type” and “Exon Splicing Complexity”, both of which require quantification of ChIP signal at splice junctions. Briefly, exon splicing type is based on the classification of exon behavior according to transcript level annotation, as described in [15]. Specifically, each exonic region is classified into six different types: promoter, constitutive (non-alternatively spliced exons), variant, alternative acceptor (altAcceptor), alternative donor (altDonor) and polyA. The ChIP-Seq signal distributions around the splice sites are then compared between alternatively spliced exons and constitutive exons to uncover significant associations. Alternatively, exon splicing complexity is based on defining exon complexity as the total number of distinct exons to which the test exon is connected (spliced reads). We used classical statistical models and permutation methods to quantify the association between splice-site localized ChIP-Seq signal and exon complexity. The two approaches differ in that the first analysis depends on a fixed set of transcript annotations, while the second analysis depends on the convergence of ChIP-Seq and RNA-Seq data and is independent of transcript annotation. Finally, our approach required the incorporation of controls for confounding factors, such as gene expression level, gene size, exon location, and other factors which may be independently associated with histone modification patterns and alternative splicing.

Our findings indicate specific histone marks are associated with exon type and splicing complexity in brain reward region. The enrichment of each histone mark varies with respect to exon type, with H3K36me3 showing the greatest enrichment at alternative isoforms relative to other marks. Random forest and permutation test show specific histone marks, such as H3K36me3 and H3K4me1, play a significant role in alternatively splicing. The computational methods developed in this study can be applied to other model organisms.

## Results

### Specific HPTMs are uniquely associated with different exon alternative splicing types

**Fig 1:**
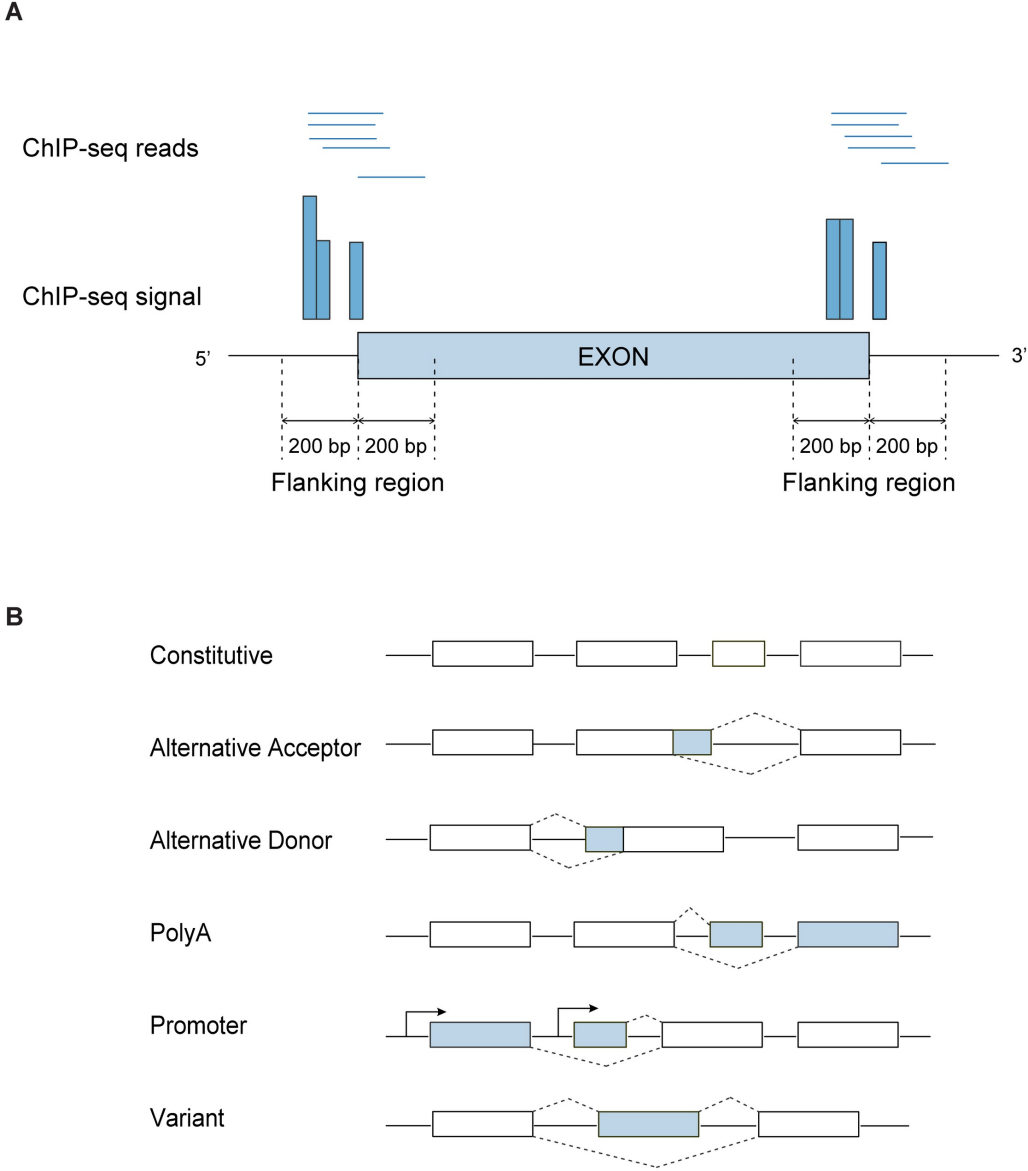
A. ChIP-Seq signal on flanking regions. ChIP-Seq signal is calculated as the number of ChIP-Seq reads (start position of reads) that aligned to each individual position of flanking regions. B: A schematic representation of different type of alternative splicing exons.

**Fig 2:**
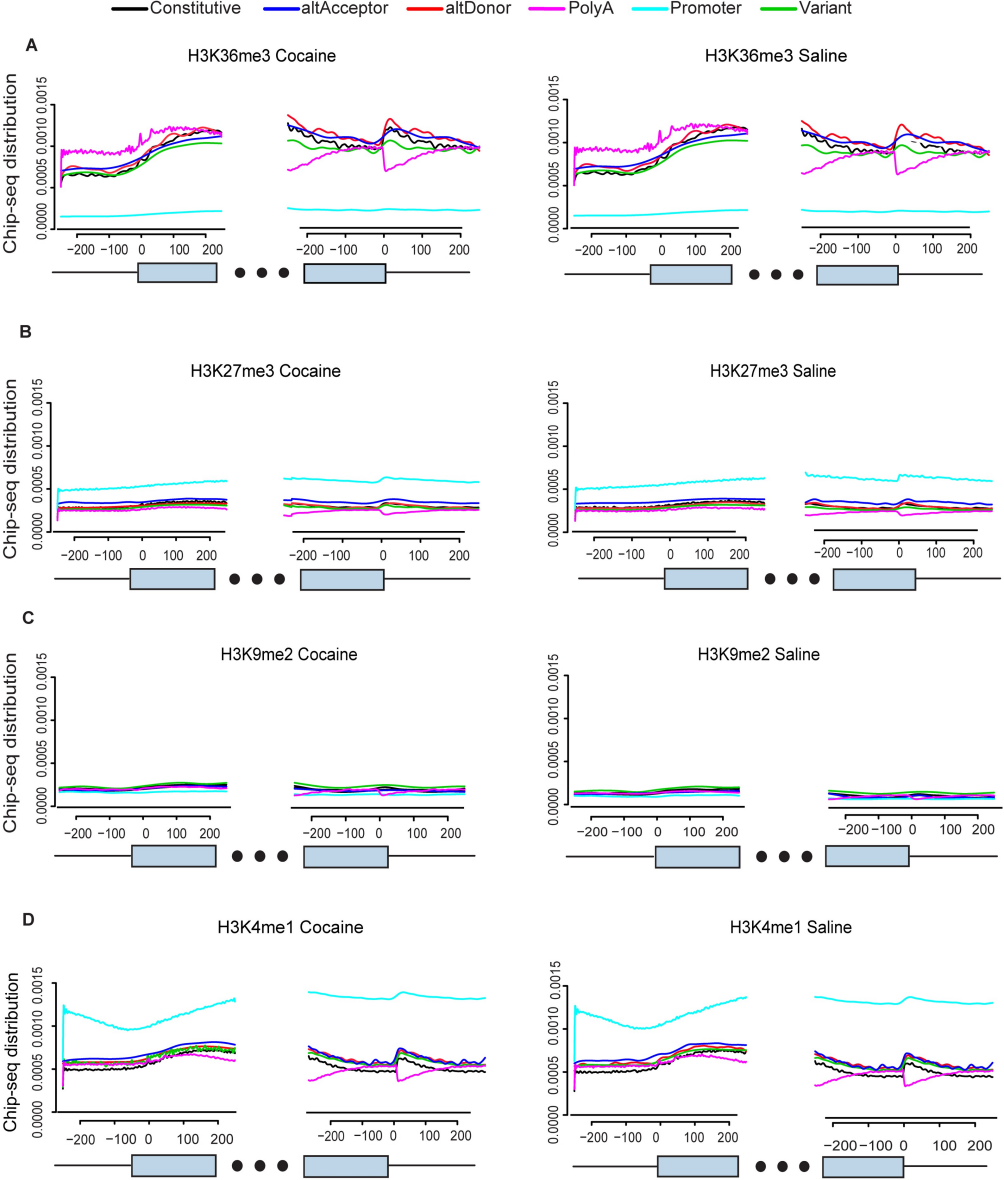
Distribution of ChIP-Seq signal on +/- 200 bp flanking regions of different exon types for four histone marks: (A) H3K36me3, (B) H3K27me3, (C) H3K9me2 and (D) H3K4me1.

We analyzed RNA-Seq and ChIP-Seq data derived from the nucleus accumbens of mice treated with cocaine (20 mg/kg i.p.) or saline for 7 days [15]. The sequences were aligned to mouse genome (mm9). The ChIP-Seq signal distributions were derived from the +/- 200bp flanking regions surrounding the acceptor and donor splice sites (Fig 1A), respectively. We then plotted the mean ChIP-Seq signal distributions for four types of histone H3 modifications under cocaine and saline (Fig 2) treatment. These include H3 lysine 36 trimethylation (H3K36me3), H3 lysine 27 trimethylation (H3K27me3), H3 lysine 9 dimethylation (H3K9me2) and H3 lysine 4 monomethylation (H3K4me1). All of these marks are differentially regulated genome-wide by cocaine administration [15,20–22]. Exon type was defined using the same criteria described in [15] based on ensemble annotation (see methods). In total there are six exon types: promoter, canonical, alternative acceptor, alternative donor, variant and polyA (Fig 1B).

As detailed below, we find that the enrichment of each histone mark varies with respect to exon type. Notably, H3K36me3 shows the greatest enrichment at alternative isoforms relative to other histone marks, with the ChIP signal being very strongly associated with alternative promoter usage (*p*-value < 2.2e-16), as compared to the other alternative splice types. In addition, H3K36me3 and H3K4me1 show the clearest separation of signal distribution patterns for different exon types, compared to the other histone marks analyzed.

The flanking regions of exons were tested for a significant difference in ChIP-Seq signal between the different non-constitutive exon types and constitutive exons, using *T*-tests. The difference is considered statistically significant if the adjusted *p*-value is smaller than 0.05. H3K36me3 is significant for alternative promoter and alternative polyA exon types, relative to constitutive exons, but only associated with some regions of altDonor, altAcceptor and variant types (Fig 2A, Table 2). Alternatively, we find that, relative to constitutive exon enrichment, the level of H3K4me1 is significantly higher in all alternatively spliced exon types except altDonor and altAcceptor at 3’ downstream (Fig 2D, Table 2), while AltAcceptor at 3’ downstream is specifically enriched for H3K27me3 and H3K9me2 (Fig 2B, Table 2). H3K27me3 also has a weak association with altAcceptor exons at 5’ and 3’ upstream (Fig 2B, Table 2). Finally, we find that the level of H3K9me2 is associated with alternative promoter and polyA exons and that there is a association with altDonor and altAcceptor exons (Fig 2C, Table 2).

**Table 2:** Differential enrichment of HPTMs at splice junctions between alternatively spliced exon and constitutive exon. *p*-value is corrected by Benjamini-Hochberg method. Significant p values are highlighted in blue [23].

Histone marker	Comparison	5’ downstream	5’ upstream	3’ upstream	3’ downstream
H3K27me3	promoter	1.91E-85	2.35E-100	9.84E-157	1.74E-111
	altDonor	0.79	0.73	0.59	0.93
	altAcceptor	0.15	0.03	0.05	0.15
	variant	0.10	0.37	0.53	0.10
	polyA	2.00E-18	5.26E-10	4.20E-23	1.20E-65
H3K36me3	promoter	0	0	0	0.00
	altDonor	0.100	0.02	0.00	0.37
	altAcceptor	0.817	0.01	0.02	0.94
	variant	0.064	0.70	0.05	0.00
	polyA	2.19E-18	4.00E-118	1.75E-32	0.00
H3K4me1	promoter	0	0	0	0
	altDonor	0.05	0.00	0.00	0.11
	altAcceptor	0.00	0.00	0.00	6.79E-05
	variant	0.07	0.00	0.00	0.30
	polyA	0.01	1.23E-07	9.75E-10	2.02E-131
H3K9me2	promoter	3.08E-23	6.11E-20	4.10E-13	1.76E-22
	altDonor	0.07	0.00	0.14	0.00
	altAcceptor	0.05	0.00	0.02	0.00
	variant	0.53	0.59	0.39	0.50
	polyA	0.00	0.05	0.00	5.64E-25

**Fig 3:**
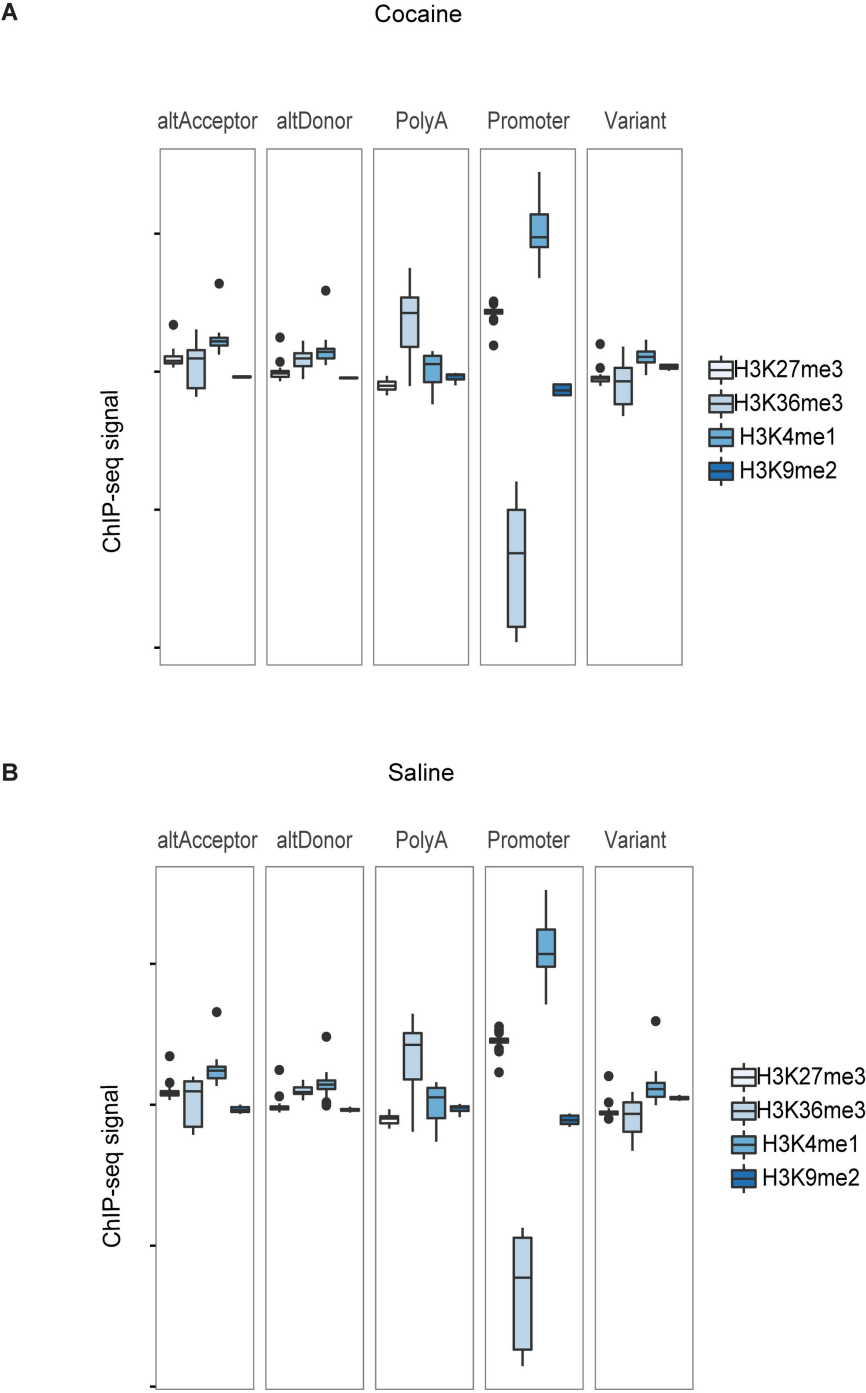
The difference between alternatively spliced exon and constitutive exon in (A) Cocaine and (B) Saline treatments.

To further explore the histone modification patterns for different types of alternatively spliced exons, we computed the difference in ChIP signal between every exon and its corresponding constitutive exon, pooled by exon type (Fig 3). We found a significant difference in ChIP signal for each exon type analyzed (*p*-value < 2.2e-16). Moreover, H3K36me3 shows the greatest variation among different exon types while there is minimal variation for H3K9me2 and H3K27me3. In summary, ChIP signal is considerably associated with alternative exon type, with H3K36me3 and H3K4me1 most strongly associated among the four histone marks studied.

### Random forest modeling predicts exon type from ChIP signal pattern

While our first analysis found that ChIP signal distribution is different for different exon types, indicating that ChIP signal can be used to identify exon types. We use Random Forests [24] to determine if the ChIP signal distributions are specific for each exon type. Random forest is an ensemble method that combines the results of multiple regression trees [24] and generally shows a better predictive performance over individual algorithms [25]. We regarded the exon type as the response variable of the model and the ChIP signal in the upstream and downstream +/- 200 bp flanking regions from exons of different exon types as the explanatory variables. This was done independently for the two treatments cocaine and saline. Table 3 shows the performance of the models based on cocaine and saline treatment do not differ significantly. The model has very good accuracy and macro-averaged precision for both treatments, with accuracy much higher than random (~0.58) (Table 3). These results demonstrate that the model has excellent power to distinguish different exon types based on the ChIP signal, indicating that the histone modifications patterns are associated with alternative splicing. In addition, the model performs equally well under cocaine and saline treatment, suggesting that chromatin-directed alternative splicing is a basal transcriptional mechanism.

**Table 3:** Performance of random forest model for cocaine and saline treatment measured by 5-fold cross validation.

	Cocaine	Saline
Accuracy	0.79	0.80
Macro-averaged Precision	0.86	0.85
Macro-averaged Recall	0.40	0.41

### Model validation that specific histone marks play a dominant role in alternatively splicing

To elucidate which histone mark is most strongly associated with the different exon type, we built the model based on the variables from each mark and further applied a process similar to the model selection approach in which each explanatory variable was progressively added into the current model to search for the best model. The model performance was calculated based on the component combination of histone marks. As illustrated in Table 4, the accuracy of models varies from 0.59 to 0.72. H3K36me3 and HK9me2 have the largest and smallest accuracies, respectively. The model performance increases with each mark added sequentially until the accuracy of model with H3K36me3+H3K4me1+H3K27me3 which is comparable to the full model—adding H3K9me2 does not further contribute to the model performance.

**Table 4:** Accuracies of random forest models built on different HMs and combination of HMs.

Variables in model	Cocaine	Saline
H3K27me3	0.66	0.66
H3K4me1	0.70	0.72
H3K9me2	0.59	0.59
H3K36me3	0.72	0.71
H3K36me3+H3K4me1	0.78	0.79
H3K36me3+H3K4me1+H3K27me3	0.79	0.80
Full Model (H3K36me3+H3K4me1+H3K27me3 + H3K9me2)	0.79	0.80

**Fig 4:**
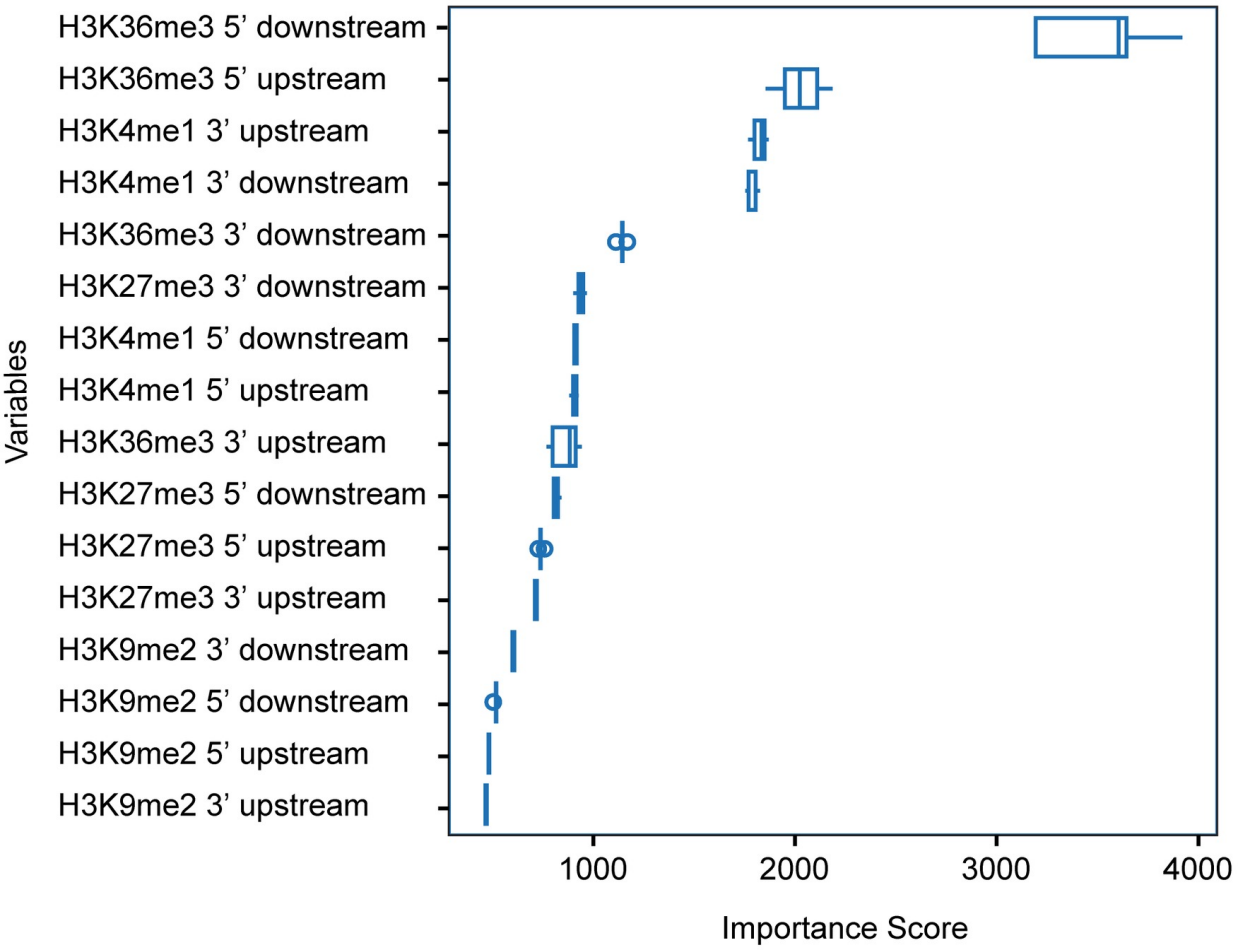
Importance score of variables from random forest model.

Additionally, we consider the importance score of each variable from the full model (Fig 4). Consistent with what we observed above, the ChIP-Seq signal of H3K36me3 in the flanking region 5’ splice site downstream is the most informative for classifying the exon type, followed by H3K4me1 at the 3’ site. The 3’ downstream of H3K27me3 at donor splice sites also shows a greater contribution than the other markers, while H3K9me2 and H3K27me2 in most of the regions are the least informative. Therefore we conclude that the H3K36me3 and H3K4me1 play a dominant role on the regulation of alternative splicing.

### Specific histone marks are significantly associated with alternatively exon complexity

**Fig 5:**
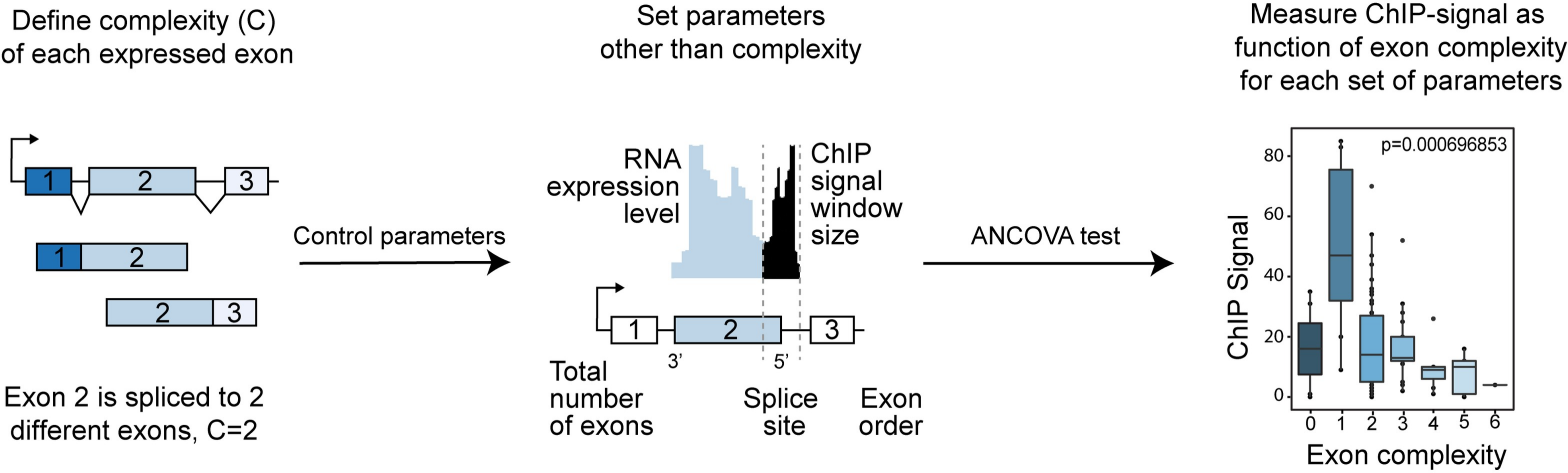
Schematic of exon complexity analysis. Exon complexity is defined as the number of distinct locations that are connected to either end of an exon, as measured by spliced reads. An algorithm was developed to rigorously control for variables other than splicing complexity that may confound our findings, including number of exons, exon order, ChIP-signal window size and gene expression level. Analysis of Covariance was used to measure ChIP-Seq signal among different exon complexity levels.

Unlike the above approach, which depends on a fixed set of transcript annotations and ChIP-Seq data, the second analysis is based on defining the exon splicing complexity as the total number of distinct exons that the exon in question is connected to by spliced reads. We test for significant association between the continuous ChIP-Seq variable and the discrete complexity variable. In testing for this association, particular attention must be made to control for the possibility of confounding factors (Fig 5).

**Fig 6:**
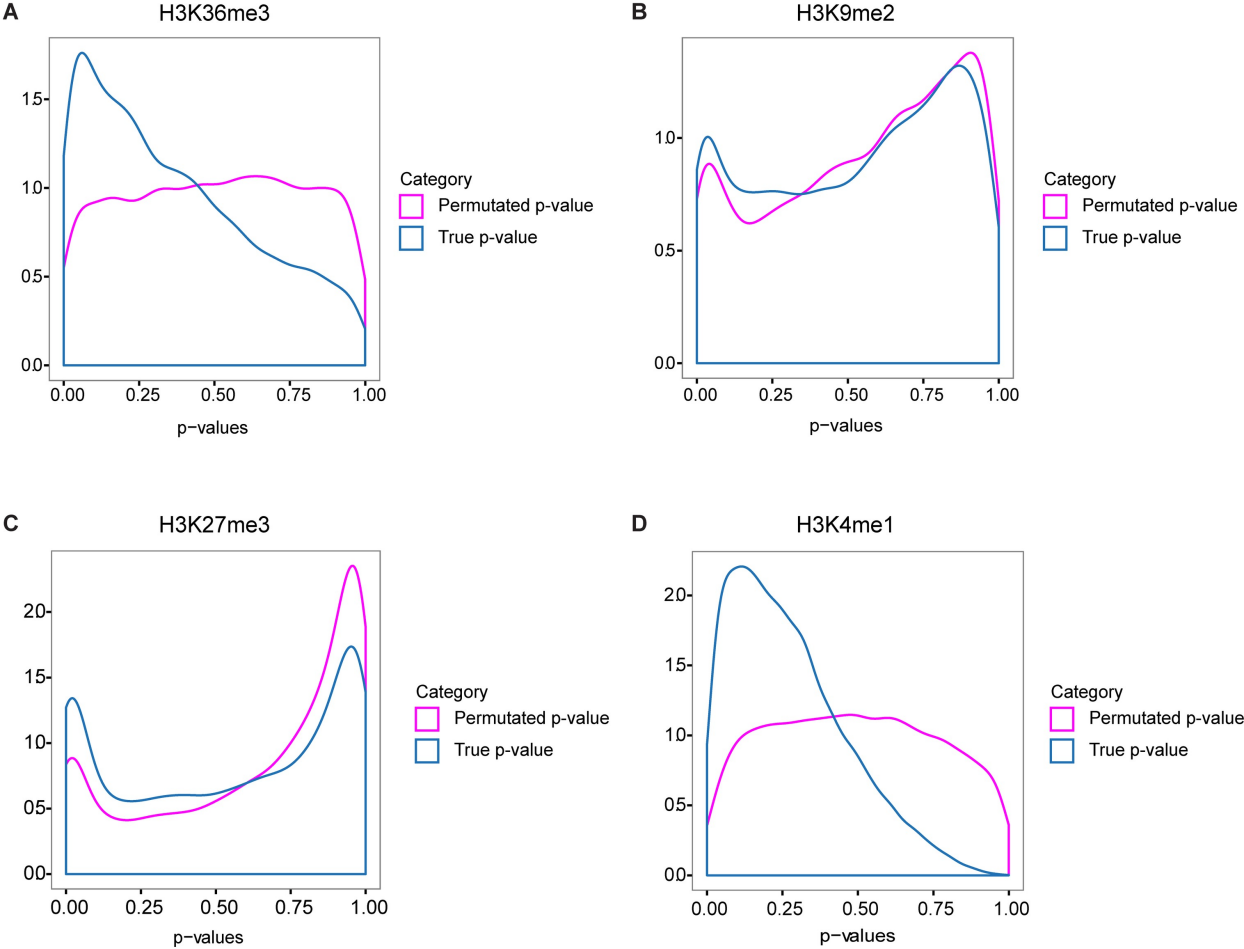
The distributions of *p*-vaues across the parameter space, for the data and for permuted controls, for four histone marks: H3K36me3 (A), H3K9me2 (B), H3K27me3 (C) and H3K3me1 (D).

ChIP signal and complexity may independently be associated with expression level, which could result in a significant association due entirely to this confounding factor. To control for this an ANCOVA model is used to regress out the expression level factor. Additionally, the association may also be confounded with the number of exons of the gene, the size of the gene, the location of the exon in the gene, the ChIP signal window size, and which samples were paired. To avoid these confounding factors, subsets of data points were chosen so as to hold all of these variables constant. We did not hold expression level constant because that would not allow for enough data points to perform the analysis, which is why ANOCVA was used to control for that factor. Since there is no clear systematic choice for the remaining parameters, we took a global approach as follows. For a fixed choice of parameters (e.g. genes with five exons, focusing on the 3’ junction of exon 2, with ChIP window size of 250 bases, pairing ChIP sample 2 with RNA-Seq sample 3) we compute the ANCOVA *p*-value for association between the ChIP signal and complexity. We plot the distribution of the ANCOVA *p*-values over the entire parameter space resulting in a distribution of *p*-values. As control, for each choice of parameters we permute the ChIP signal randomly among the genes and compute the ANCOVA *p*-value for the permuted data. We plot the distribution of these permuted ANCOVA *p*-values over the entire parameter space for comparison to the distribution of unpermuted *p*-values. A significant separation of these distributions, particularly near the small values of *p*, indicates a significant association between ChIP signal and splicing complexity. This approach of looking for a separation of real and permuted distributions over the entire parameter space avoids making an arbitrary choice of parameters. We see a clear separation of distributions for two of the histone marks, H3K36me3, and H3K4me1 (Fig 6). This is consistent with prior molecular biological approaches demonstrating that these two marks are functionally involved in alternative splicing (see Discussion). This finding is also consistent with that derived from the exon type analysis above, indicating that these two histone marks are specifically associated with alternative pre-mRNA splicing in in the brain.

## Discussion

Alternative isoform expression presents a compelling mechanism by which neurons mount a stable response to environmental stimuli, functionally analogous to stable isoform selection by differentiating neurons during development. In the latter context, alternative splicing confers neuronal identity and is maintained throughout life [26]. We thus sought to explore the epigenetic regulation of alternative splicing through an investigation of the genome-wide association of specific histone modifications and alternatively expressed exons. To uncover the genome-wide association between histone modifications and alternative splicing in mammalian brain, we analyzed global, exon-level splicing patterns in neurons. We designed two approaches to test for the association of specific alternative splicing patterns and histone modifications, in order to determine which play a significant role in the regulation of alternative pre-mRNA splicing.

We first developed a method to demonstrate the association of particular histone modifications with different types of alternatively spliced exons in brain. This led to the finding that enrichment of each analyzed histone modification varies with respect to each type of spliced exon, with H3K36me3 showing the greatest enrichment at alternative isoforms relative to other histone post-translational modifications. This result is well founded in the context of prior research in non-neuronal systems. First, genome-wide analyses of nucleosome-positioning data sets from humans, flies and worms show that exons have increased nucleosome-occupancy levels with respect to introns [6,27], and that H3K36me3 is found consistently to be preferentially enriched in exons. Second, the correlation of exon inclusion levels and nucleosome distribution patterns suggests that nucleosome positioning defines exons at the chromatin level, indicating that DNA-coded splicing signals mediate the observed differences in the chromatin landscape of exons and introns. Finally, beyond nucleosome occupancy, exons are differentiated from introns by specific histone modifications [7,28], which may play a key role in exon recognition during co-transcriptional splicing.

**Fig 7:**
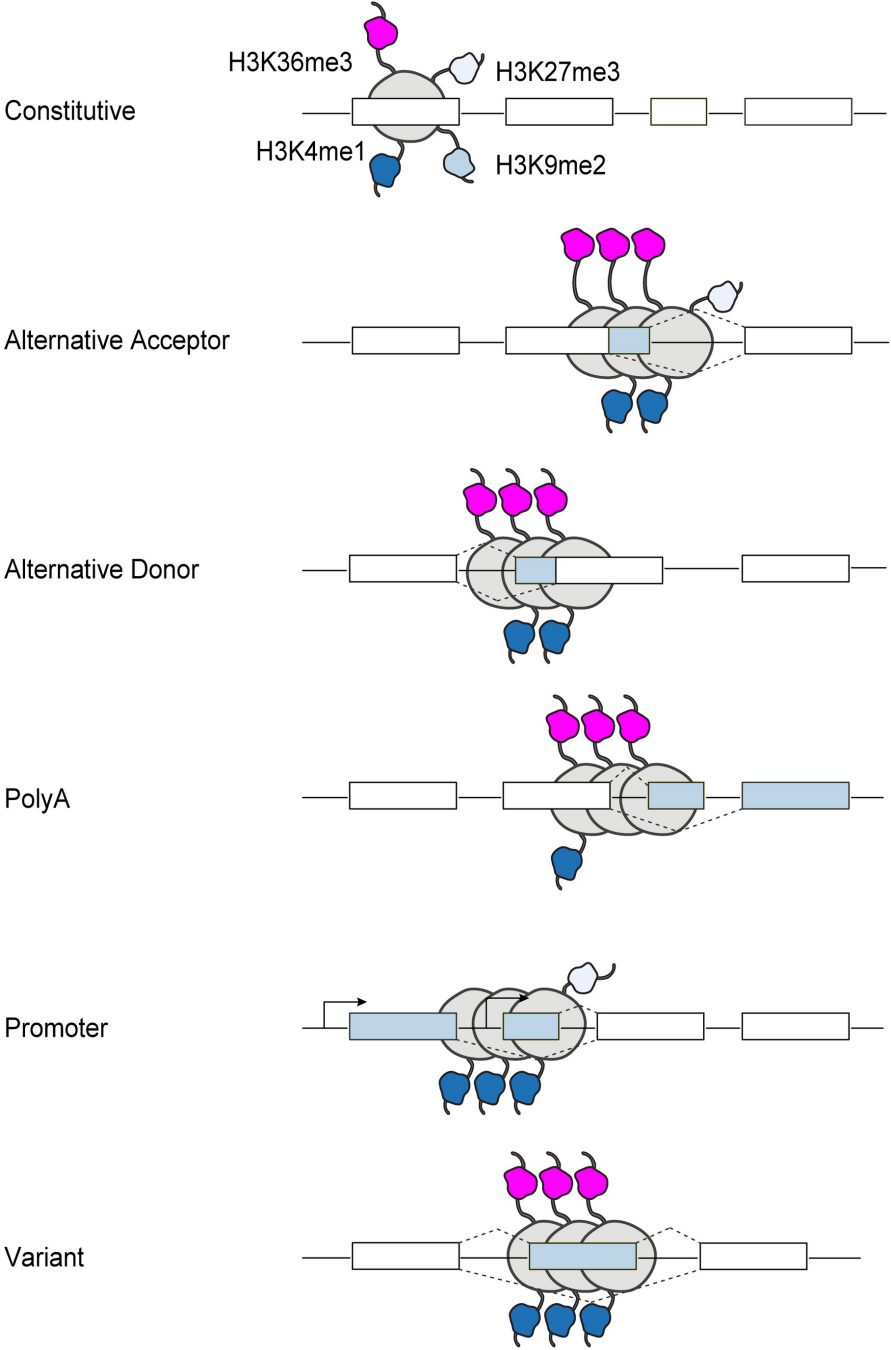
Contribution of histone modifications to the regulation of alternative splicing. Unbiased global analysis reveals that the enrichment of specific histone marks varies with type of alternatively spliced exon. H3K36me3 and H3K4me1 show a much stronger association with alternative splicing than H3K27me3 and H3K9me2. H3K36me3 is maximally enriched at alternative PolyA exons, while at promoters it is depleted and H3K4me3 is maximally enriched.

We took an unbiased approach to examine the behavior of histone modifications at alternative splice sites, and discovered that H3K36me3 and H3K4me1, but not H3K9me2, show a clear separation of distribution patterns for different types of alternatively spliced exons. Specifically, we find that H3K36me3 is highly significantly associated with alternative promoter and alternative polyA splice types. Furthermore, the ChIP-Seq signal of H3K36me3 in the flanking region of the acceptor splice site is the most informative for classifying alternative exon types using random forest classifiers (Fig 7). These results are especially promising given prior data using a *de novo* pattern-finding algorithm which indicates that enrichment of H3K36me3 correlates with increased exon usage in alternatively spliced genes [14]. This finding was expanded in a study in *C*. *elegans*, which further confirmed that the exon enrichment of H3K36me3 is globally conserved in human and mouse genomes [29]. While ours is the only genome-wide computational analysis to associate H3K36me3 with alternative splicing in a mouse brain reward region, several groups have reported promising experimental data to support this mechanism at specific genes. For example, using a β-globin gene reporter system, Kim et. al. demonstrated that splice-site mutations, which correlated with enhanced retention of a U5 snRNP subunit on transcription complexes downstream of the gene, affected H3K36 methylation, while a polyA site mutation did not [5]. Further, global inhibition of splicing by spliceostatin A caused a rapid repositioning of H3K36me3 away from 5’ ends in favor of 3’ ends, indicating a direct relationship between splicing mechanisms and H3K36 methylation status. Finally, a landmark paper by Luco et. al. demonstrated a direct interaction between H3K36me3 and the spliceosome machinery, specifically PTB and MRG15, at the FGFR2, TPM2, TPM1, and PKM2 loci in human cell lines [4]. Together with the results of our global analysis, these studies strongly implicate H3K36me3 in mediating alternative splicing *in vivo*.

As described above, the role of specific histone modifications in marking exons is well-documented, yet there is limited data on this mechanism specifically in the brain, despite the critical role of alternative splicing in neuronal identity and survival [18,26,30]. Our prior study on the role of histone modifications in the nucleus accumbens found that, compared to saline, cocaine drives differential isoform expression to a markedly greater extent [15]. Further, particular histone modifications were differentially enriched by cocaine treatment, and this enrichment could be further distinguished on the basis of the associated exon alternative splice type. To expand upon this previous finding, we focused on an exploratory analysis of the relationship between histone modifications and specific types of alternative splicing and developed a systematic approach to test for the global association between them. Specifically, while the exon type annotation is similarly derived, the exon type approach differs from our prior analysis in that we model the association based on the distribution, but not enrichment between treatments, of histone marks in flanking regions. The association we derived is therefore a global effect and independent of specific target clusters within treatment groups (see methods). Despite these distinct computational approaches, both studies identify an important role of H3K36me3. With respect to differential enrichment, Feng et. al. found that this mark is differentially enriched in cocaine-treated NAc tissue at alternative donor, alternative acceptor and variant spliced exon types, all of which we find here to be significantly differentially enriched for H3K36e3 relative to constitutive exons. Similarities in the two datasets also emerge with the analysis of H3K4me1 and me3, which are found to be differentially enriched in cocaine-treated samples at alternative acceptor and variant exon types [15]. While our previous analysis identified a clear role for H3K4me3 in the association with alternative exon expression, our current method was unable to analyze this particular mark due to insufficient read coverage of ChIP-Seq samples necessary for the complexity analysis. Finally, H3K4me1 and H3K4me3 enrichment has also been implicated in chromatin-directed alternative splicing in cell culture [4], indicating a functional role for both H3K4me1, H3K4me3 and H3K36me3 in alternative splicing.

We have taken an unbiased approach to investigate chromatin-directed alternative splicing in brain, having developed an innovative computational model to test the association between alternative exon expression and specific histone modifications. Using this method we have applied a single statistical test to the association of ChIP-Seq and RNA-Seq data within a brain-derived dataset to find that there are highly significant associations between alternative splicing and the specific histone marks H3K36me3 and H3K4me1. This association is found in both treated and un-treated neuronal tissue, indicating the fundamental nature of this global mechanism. Future studies will be needed to discern the role of cocaine, as well as other forms of neuronal activation, in the regulation of chromatin-directed alternative splicing, both globally and at specific genes.

## Methods & models

### ChIP-Seq/RNA-Seq data and preparation

High-throughput ChIP-Seq and RNA-Seq datasets from [15] were downloaded from GEO (https://www.ncbi.nlm.nih.gov/geo/, GSE42811). Please refer to the published work for details on animal treatment and sample preparation [15].

For each treatment, four histone modifications were assayed: H3K36me3, H3K4me1, H3K27me3 and H3K9me2 using ChIP-Seq. ChIP-Seq reads were aligned to mouse reference genome (NCBI37/mm9) using Bowtie2 (Version 2.1.0) with default parameters [31]. The RNA-Seq reads were aligned using STAR (Version 2.4.1d) [32] with index built with RefSeq annotation. Data were normalized and quantified by the PORT pipeline (https://github.com/itmat/Normalization) and further normalized for gene length by the FPK method (fragments per kilobase of gene length). The exon-level expression values were also normalized for read depth by PORT.

### Exon alternative splicing type analysis

The definition of exon type depends on a given set of gene annotations and is defined using the criteria described in [15]. Different exon types were classified by pairwise comparison of the boundaries of exons across various isoforms from the same gene. There are six exon types in total: promoter, constitutive, alternative acceptor, alternative donor, variant and polyA. Briefly, each gene’s exons were sorted from the 5’ to the 3’ end according to their genomic coordinates; then each exon is compared across isoforms from the same gene. If an alternative left or right boundary is found, it is classified as “alternative acceptor/donor.” If an exon overlaps with an intron, it is classified as “variant” and if there is an alternative boundary found in the first/last exon, it is classified as “promoter/polyA.” For simplicity, exons that belong to multiple types were removed from analysis. Thus each exon type represents a unique combination of exon-intron boundaries.

### Histone modification signal calculation and modeling

Histone modification signals on the flanking regions of exons were calculated in two steps: First, for each exon, flanking regions were defined as the 400bp centered at the acceptor and donor splice sites respectively. If the length of an exon is less than 400bp, the two flanking regions were truncated so they do not overlap. Second, for a given exon, we kept only the ChIP-Seq reads that overlap at least one of the two flanking regions (Fig 1A). The total number of overlapping reads was then equalized across all samples by random resampling, to make them comparable. The resampling approach results in uniform (null hypothesis) signal distributions across all samples, while scaling approaches result in uniform means but heterogeneous distributions, which is not desirable. The normalized reads were then quantified for each flanking region. To visualize the signal distribution for each exon type, we computed the *average* ChIP-Seq signal across the flanking regions, averaged over all exons of the same type (Fig 1A and Fig 2). We further divided the regions surrounding each splice site into the exonic and intronic regions with 200bp length each (except when truncated due to a small exon length). For each exon this gives ChIP-Seq signal for four regions: the intronic region at the acceptor splice site (5’ upstream), the exonic region at the acceptor splice site (5’ downstream), the intronic region at the donor splice site (3’ downstream) and the exonic region at the donor splice site (3’ upstream).

One of the ways that we demonstrate that there is an association between ChIP signals and exon type is by showing that ChIP-Seq signal is predictive of exon type. Therefore, we consider this as a multi-class classification problem, for which a variety of classification algorithms can be applied. In this study, we used the Random Forest classification algorithm because of the wide consensus on its performance [25]. Additionally, Random Forest classification provides a ranking of the different histone modifications by their predictive power.

The performance of the model was evaluated by 5-fold cross validation. The dataset was randomly partitioned into five equal sized subsamples. Among those five subsamples, one subsample was used as test data to evaluate the model performance and the remaining four subsamples were used as training data to construct the model. The model was further tuned based on training data to achieve the best parameters by calculating the model performance under different combination of parameters. The model with lowest error rate was then selected. Then Accuracy, macro-averaged precision and macro-averaged recall were calculated based on test data to measure the model performance. These values were calculated according to the confusion matrix as shown below:

$$\begin{array}{l} \mathrm{Data}\;\mathrm{class}\;\mathrm{Classified}\;\mathrm{as}\;\mathrm{Type}\;i\;\mathrm{Classified}\;\mathrm{as}\;\mathrm{Type}\neq i\\ \mathrm{Type}\;i\;\mathrm{true}\;\mathrm{positive}\;\left(\mathrm{tp}\right)\;\mathrm{false}\;\mathrm{negative}\;\left(\mathrm{fn}\right)\\ \mathrm{Type}\neq i\;\mathrm{false}\;\mathrm{positive}\;\left(\mathrm{fp}\right)\;\mathrm{true}\;\mathrm{negative}\;\left(\mathrm{fn}\right) \end{array}$$

For each exon type *i*, *i* = 1…6, the confusion matrix is calculated as:

$$Accuracy=\frac{\sum_{i=1}^{6}\frac{tp_{i}+tn_{i}}{tp_{i}+fn_{i}+fp_{i}+tn_{i}}}{6}$$

$$Error\;rate=\frac{\sum_{i=1}^{6}\frac{fp_{i}+fn_{i}}{tp_{i}+fn_{i}+fp_{i}+tn_{i}}}{6}$$

$$Macro-averaged\;precision=\frac{\sum_{i=1}^{6}\frac{tp_{i}}{tp_{i}+fp_{i}}}{6}$$

$$Macro-averaged\;recall=\frac{\sum_{i=1}^{6}\frac{tp_{i}}{tp_{i}+fn_{i}}}{6}$$

Five-fold validation means this whole process was repeated five times, with each of the five subsamples used as the test data in turn.

### Exon splicing complexity analysis

Splicing complexity is an integer associated to the 5’ or 3’ end of an exon. In contrast to exon type, complexity depends on a particular RNA-Seq data set. In particular for a given exon boundary *b*, the complexity *c(b)* is the number of distinct *locations* that are connected to location *b* by a spliced read. For a fixed location *x*, there may be many reads which splice from *b* to *x*, however the complexity only counts distinct locations, not distinct reads, so each distinct genome location *x* increments the complexity by one if there are any reads at all spliced from *b* to *x*; otherwise it increments by zero. The splice junction indicated by the read may or may not be annotated; all reads which splice from location *b* are counted. For example, if a gene has only one expressed splice form, then the complexity of all of its exon boundaries equals one, except at the two boundaries consisting of the start and end of transcription, for which the complexity equals zero. With few rare exceptions, complexity typically varies from between zero and ten.

In this analysis a statistical association between splicing complexity and ChIP-Seq signal is tested for. We expect splicing complexity to increase with the expression level of the gene, which could then be confounded with the ChIP signal, if that signal is also associated with expression level. An ANCOVA model is employed to control for this possibility. The ANCOVA model can be formulated as follows:

$$y_{ij}=\mu +\alpha_{i}+\beta x_{ij}+\epsilon_{ij}$$

Where *y*_*ij*_ is the ChIP-Seq (histone modification) signal of exon *j* with splicing complexity *i*, *x*_*ij*_ is the normalized FPK (expression level) of exon *j* with splicing complexity *i*, *μ* is the reference level, *α*_*i*_ is the effect of splicing complexity *i* (*i* = 1….*n*), and *β* is the regression slope that quantifies the (linear) relationship between the FPK and the ChIP-Seq signal.

Type III SS is used for testing the significance of each splicing complexity level (Ho: *α*_1_ = *α*_2_ = *α*_3_ = … = *α*_*n*_ = 0) and the linear relationship between the ChIP-Seq signal and FPK (Ho: *β* = 0). If the test is significant, it indicates that after controlling for expression level, there is still a significant difference for the ChIP-Seq signal among different exon complexity levels.

## Data access

High-throughput ChIP-Seq and RNA-Seq datasets from [15] were downloaded from GEO (https://www.ncbi.nlm.nih.gov/geo/), accession number GSE42811.
